# Use of Genomic Tools to Improve Cattle Health in the Context of Infectious Diseases

**DOI:** 10.3389/fgene.2016.00030

**Published:** 2016-03-07

**Authors:** Mikolaj M. Raszek, Le L. Guan, Graham S. Plastow

**Affiliations:** Livestock Gentec, Department of Agricultural, Food and Nutritional Science, University of AlbertaEdmonton, AB, Canada

**Keywords:** next-generation sequencing, cattle infection, pathogen genome, robustness, bovine microbiome, bovine virome, bovine respiratory disease, breeding programs

## Abstract

Although infectious diseases impose a heavy economic burden on the cattle industry, the etiology of many disorders that affect livestock is not fully elucidated, and effective countermeasures are often lacking. The main tools available until now have been vaccines, antibiotics and antiparasitic drugs. Although these have been very successful in some cases, the appearance of parasite and microbial resistance to these treatments is a cause of concern. Next-generation sequencing provides important opportunities to tackle problems associated with pathogenic illnesses. This review describes the rapid gains achieved to track disease progression, identify the pathogens involved, and map pathogen interactions with the host. Use of novel genomic tools subsequently aids in treatment development, as well as successful creation of breeding programs aimed toward less susceptible livestock. These may be important tools for mitigating the long term effects of combating infection and helping reduce the reliance on antibiotic treatment.

## Introduction

Infectious disease is a major economic burden in the production of cattle worldwide. Cattle aﬄicted with pathogenic infections incur treatment costs, deliver reduced performance, lower yield grades, increased mortality rates and can lead to reproductive loss (Chi et al., 2002; Snowder et al., 2006; Garcia et al., 2010). Bovine respiratory disease, a complex involving different pathogens and contributing factors, is a persistent and leading cattle health problem in North America and the world (Snowder et al., 2006; Garcia et al., 2010) with an estimated negative annual economic impact of $3 billion (all currency in U.S. funds; Griffin, 1997; Srikumaran et al., 2007). In the U.S., BRD accounts for ∼75% of feedlot morbidity and ∼57% of all feedlot deaths (Edwards, 1996; Loneragan et al., 2001). A recent estimate suggests more than 4 million feedlot cattle were affected by BRD in 2013 (Neibergs et al., 2014a). In the case of dairy, historical data indicates that mastitis can lead to annual loses of $1-2 billion in U.S. dairy industry (Petrovski et al., 2006). The annual cost of lost production and control of nematode parasites has been estimated at over $2 billion per annum for the North American cattle industry with even greater losses in many parts of the world (Stromberg and Gasbarre, 2006). Other common diseases of dairy farms, BVDV, Johne’s disease, and nesporosis, have been estimated to cause annual losses of ∼$2,500/50-cow herd, while enzootic bovine leukosis was estimated to lead to ∼$800/herd production loss (Chi et al., 2002). These values indicate the staggering economic impact associated with pathogenic infection.

BRD also exemplifies the typical compounding factors that impose limitations on disease management in general. Currently there is no cure for BRD, and vaccines have a 50–70% success rate in prevention (Dabo et al., 2007; Rice et al., 2007). Outbreaks are difficult to diagnose in the field, and even epidemiologic studies are typically inconclusive in defining the exact agent responsible for development and progression of the disease as isolation of multiple bacterial and viral pathogens is common (Dabo et al., 2007).

In addition to the direct impacts of mortality and morbidity, further cause of concern is the scale of antimicrobial use in the livestock industry, and its potential repercussions on public health (Bush et al., 2011). Volumes of antimicrobial distribution by manufacturers indicate that agricultural use can exceed human antibiotic needs by more than eightfold (Government of Canada, 2011). Of those, ∼90% are used to promote growth or prophylactic protection rather than infection treatment, factors that advance microbial resistance and seriously undermine ability to treat common infectious diseases (Government of Canada, 2013). Most classes of animal drugs are also used in humans, and transfer of resistance through zoonotic infections can have adverse effects on human health (Government of Canada, 2003). Ironically, emerging calls for reduced usage of antimicrobials in agriculture could lead to reduced efforts to develop these products (Bush et al., 2011; Ontario Medical Association, 2013). It is also possible that the recent rise in public suspicion of vaccine use could also have an adverse impact on its use in food animals, especially considering the growing trend of consumer interest in healthy animal products (Dube et al., 2013; Egger-Danner et al., 2015). Such developments will only add to the existing strain of effective farm health planning.

Against this background what are the most practical methods of maintaining animal health? If we assume that drug treatments will become less acceptable then what are the alternatives? There is an array of potential solutions, however, perhaps two of the most attractive are the breeding of animals that are naturally robust (defined here as the ability to tolerate departure from an optimal environment, such as infections, with minimal fitness impact) together with improved animal management supported by better diagnostics and targeted treatment. Both of these solutions will benefit from a deeper and fundamental understanding of how animal health is impacted by host genetics, environmental factors, and microbial infection.

Success of breeding or managerial programs are acutely reliant on the measurement accuracy of health related phenotypes. Assessment of infection or disease development requires confirmation of which organ systems are being affected, and what etiologic agents are involved. Multiple diagnostic tests have been developed for such assessment, including serological tests, pathogen isolation, antibody staining, and, more recently, molecular based approaches that rely on amplification and detection of nucleic acids (Fulton and Confer, 2012). While they cannot be relied upon solely for diagnosis, next-generation sequencing technologies offer the newest approaches to etiological studies of infectious diseases in cattle because they can detect multiple pathogens and the genetic expression patterns of pathogens. The genetic expression reaction of the host can also be monitored via high throughput sequencing, as well as the observation of genomic differences between bovine hosts in relation to the observed disease phenotype.

This review examines the use of high throughput data delivery of genomic tools to protect cattle from infectious diseases, a technology that increases our understanding of host/pathogen interactions, improves diagnosis, enhances breeding programs, and enables treatment development. Specific examples in the beef and dairy sectors are provided.

## Overview of Genomic Tools

The great advantage of modern high throughput sequencing is an ability to rapidly amass a large quantity of genomic information. In the cattle industry this technology can gather genetic, transcriptomic, and epigenetic information from the cattle (host), its microbiome and virome, as well as the invading pathogens. Bioinformatic metagenomic analysis of such data can help to elucidate the interplay between the host, microbiome, virome and the pathogens and the resulting disease phenotype which can enrich understanding of pathogenicity and disease etiology. This is achieved through (1) the discovery of novel information leading to enhanced structural and functional annotation, and (2) comparison of many organisms allowing researchers to deduce differences between different states.

Rapid gains in pathogen genomic data are leading to the discovery of novel infectious agents as well as to a more refined subdivision of known pathogens. A study of the pathogen transcriptome can decipher virulence factors and elucidate both intrahost and interhost pathogenicity on a wide geographic scale. This information can aid in the diagnostic quest and influence the management of animals to reduce both the pathogen source and pathogen transmission. Detailed genetic understanding of pathogenicity can be utilized in treatment development, including reverse genetic engineering, the appropriate selection of whole organisms to produce prophylactic treatments such as vaccines and probiotics, and to alter existing treatment strategies.

The host transcriptome analysis can provide information on expression changes in relation to pathogen challenge, or treatment, which can provide diagnostic biomarker information. Next-generation sequencing has uncovered 1000s of point mutations across the bovine genome, termed SNPs, structural variation, as well as epigenetic impacts. Besides probing for epigenetic DNA modifications directly, detailed comparisons between the host genome and its expression between parent and offspring can provide clues to important imprinting patterns. These genomic alterations can be collectively used in a training population of animals with clearly defined health and disease phenotypes in GWAS and genomic selection (see below). GWAS can yield QTL information that links segments of the genome to a phenotype of interest, and can develop a prediction of the genetic merit in the next generation, including complex polygenic traits. Such genetically enhanced EBVs enable faster and more accurate genetic improvement compared to conventional methods, at a low cost of inbreeding rate. Knowledge of which genetic components take part in disease progression or resistance can be used to breed more robust animals. It can also be used for host genomic engineering, as technologies now exist that permit this approach in a site specific manner (gene editing, see below).

The genome and transcriptome of the host microbiome and virome can be analyzed in a similar fashion to decipher the potential influence of each on health and disease, with a future goal of precise manipulation to manage animal health.

Finally, the process of discovery of genomic information propagates development of additional validating technologies that can be used in genotyping for specific information, and aid in cumulative knowledge progression. Prior data can be used in the design of sensitive microarrays, as well as for imputation purposes, increasing the power and resolution of analysis studies.

## Pathogen Identification and Disease Profiling

The rapid and large data output of next-generation sequencing can enable detailed and broad identification of disease pathogens. Pathogen genetic makeup and expression can throw light on (1) disease diagnosis, and (2) the pathogenicity of the infectious agent. In turn, understanding of disease etiology can be improved through (1) identification of the spectrum of infectious agents involved, including novel information, (2) surveillance of geographical disease transfer, and (3) identification of a reservoir source. For example, a contagious udder infection in a Holstein–Friesian herd in Poland was identified, in part by sequencing, to be caused by *Staphylococcus microti*, a rarely observed microorganism and the first instance reported to be a causative agent of mastitis, or its presence in domesticated animals (Krol et al., 2016). The reservoir source has not yet been confirmed, although rodents as possible carriers were suspected.

Sequencing can prove useful in studying the etiology of diseases, such as the BRD complex, that are poorly understood due to the number of pathogens involved. Clinical symptoms from studies of single pathogen infections do not necessarily replicate what is observed in the field (Sarrazin et al., 2014). In a recent study the coexistence of multiple pathogens was found in 97% of BRD mortalities in cattle from feedlots in Alberta, Nebraska, and Texas. 16.2% of cases identified co-occurrence of three pathogens, while in 11.8% of cases co-occurrence of four pathogens was observed (Klima et al., 2014b). The pathogens most frequently associated with BRD were *Mannheimia* (*Pasteurella) haemolytica* (91%), BVDV (69%), *Mycoplasma bovis* (63%), *Histophilus somni* (57%), BRSV (19%), and *Pasteurella multocida* and PI3V (13% each; Klima et al., 2014b). However, an established but rarely studied etiological agent of BRD, bovine rhinitis viruses, were shown to not only be a common pathogen (6.4% of the tested nasal swab samples), but also included strains that have previously not been observed in North America (Hause et al., 2015). These results were confirmed by another nasal swab virome analysis, also leading to discovery of some uncharacterized viruses, while demonstrating that 38% of BRD sick animals versus 8% of controls were infected with multiple respiratory viruses. Bovine adenovirus 3, bovine rhinitis A virus, and bovine influenza D virus were detected in 62% of animals with BRD, clearly demonstrating greater scope of complexity of viruses involved in the epidemiology of the disease (Ng et al., 2015). It can be expected that diseases such as BRD will be categorized into additional subtypes based on the identified pathogens, giving rise to more precise diagnoses.

An additional factor to consider is the impact of heterologous reactivity—the influence of an infection engendered by one pathogen on the outcome of an infection engendered by another pathogen. The impact of heterologous reactivity can be complex and the final effect on host health can be either negative or protective, depending on the combination of organisms involved. The potential of heterologous protection against East Coast fever (caused by infection with the *Theileria parva* protozoan) was recently explored in Kenyan calves that were co-infected with *Theileria mutans*, a species that does not lead to the severe disease outcome evinced by *T. parva*. It was shown that co-infection reduced East Coast fever mortality by more than 40% (Woolhouse et al., 2015). The most widely used vaccine against East Coast fever is in fact a cocktail of three live *T. parva* isolates. Recent sequencing of the genomes of these isolates revealed extensive differences between each species and from the isolates they protect against (Norling et al., 2015). The cumulative impact of a pathogen blend can therefore be an influencing factor on the observed variable disease epidemiology, which in turn can affect the treatment efficacy. This underscores the need of precise diagnosis of pathogens involved in the disease etiology, including an understanding of the genetic makeup that contributes to the observed phenotype.

An example of how accumulated sequencing data of pathogen genomes can aid in the diagnostic quest was shown with the analysis of 28402 SNPs of 133 different MAP genomes, the causative agent of Johne’s disease in cattle, which allowed the development of a discriminatory assay that could differentiate between the 133 genomes with the use of just 93 SNPs (Leao et al., 2015).

Geographical comparisons can reveal regional differences defining disease etiology that could provide opportunities to tailor treatment regimens based on pathogen genomic information. The largest ever dairy cattle diagnostic survey of BRD pathogens showed distinct bacterial and viral distributions between California and New Mexico populations (Neibergs et al., 2014b).

Disease tracking in a cattle population through pathogen classification is another important opportunity to use next-generation sequencing, in terms of both surveillance and disease eradication. In one example, a sequencing study of BVDV isolates responsible for an acute outbreak of bovine viral diarrhea in North America showed that these isolates belonged to the same strain of virus (Ridpath et al., 2006). Reisolation of the same strain in multiple geographical areas suggested an explosive transmission that was previously underappreciated. Furthermore, the viral strain was characterized by a genetic insertion that distinguished it from other strains, which could account for the virulence of the pathogen. The Norwegian BVDV-eradication campaign of the 1990s demonstrated that systematic monitoring of beef and dairy cattle can lead to the elimination of this economically burdensome infectious disease (Norstrom et al., 2014). The cost of the decade-long project was estimated at $8 million with a savings in potential losses of up to four times that amount (Loken and Nyberg, 2013). At the time, the viral infection was monitored by tracking antibodies in pooled herd blood. More recently PCR-based detection has been used to screen herds for carriers (Drew et al., 1999; Kennedy et al., 2006), but next-generation sequencing could be employed instead for direct pathogen identification, allowing for tracing of a much broader pathogenic burden.

Novel diseases of unknown etiology, misdiagnosed infections, and inappropriate treatments might also be uncovered via knowledge of the pathogens involved. An unusual *Mannheimia* sp. cluster V and BRSV infection in British Columbia Holstein calves aﬄicted with bronchopneumonia were identified through 16S ribosomal RNA gene sequencing when the initial diagnosis using phenotypic and biochemical procedures suggested the presence of *M. granulomatis* (Britton and Zabek, 2012). Metagenomic analysis allowed the discovery of a novel viral pathogen (G15P[14]) that was responsible for an epizootic outbreak of diarrhea in adult cows in Japan in 2013 (Masuda et al., 2014). Deep sequencing also led to a diagnosis of the cause of an unusual high mortality acute diarrhea outbreak in Germany in 2013. An unfamiliar obligatory mixture of BVDV viruses were shown to be responsible for the disease (Jenckel et al., 2014). Approximately 95% of the isolates consisted of a BVDV-2 virus containing a 222 nucleotide segment duplication within its polyprotein-encoding region; different mutations were found between the two duplicate regions. The remaining minor fraction consisted of two additional BVDV strains that contained either of the duplicate regions, and were generated *de novo* from the virus containing the duplication, demonstrating hitherto unrecognized viral genomic plasticity. Deep RNA-Seq has been utilized to identify and characterize a novel swine and bovine influenza virus in Oklahoma. Serological data suggested that cattle might represent the virus reservoir, and presence of the virus in more than one species prompted authors to call for further investigation of the potential impact of the virus on human health (Hause et al., 2014).

Novel tools provided by next-generation sequencing can be used to correlate animal health status with a specific disease. Thus sequencing can facilitate disease identification through broader understanding of the pathogens involved, as well as the discovered mutational load (observed variation in DNA of the pathogen) that impact disease progression in animals.

## Host–Pathogen Interaction and Microbiome Influence

Next-generation sequencing can be utilized to investigate molecular pathogen–host interplay via the study of pathogen and host transcriptomes. Furthermore, such molecular response analysis can be extended in the context of host microbiome and virome influences on disease development. The benefits of genome expression analysis can be translated into infectious disease management via (1) knowledge of both host and pathogen gene function in disease development and (2) biomarker development from observations of molecular outcomes in the host. The full annotation of gene function in pathogens could inform measures to improve animal health by directing future treatment development, or could be utilized in breeding programs (discussed in sections below).

For example, comparative analysis of bovine macrophage transcriptomes infected by either *M. bovis* or MAP at different time points post-infection, demonstrated much more robust immune response to *M. bovis* than MAP (Rue-Albrecht et al., 2014). The analysis of the pattern of expression suggests that the immunoevasive capabilities of MAP reside in its ability to suppress pro-inflammatory responses, and thus enabling it to reside and replicate in macrophages for prolonged periods of time, whereas that of *M. bovis* relies on enhanced type I interferon-inducible gene expression to suppress IL-1 production required for host control of infection. The use of next-generation sequencing to examine the transcriptome can provide detailed insight of such pathogen–host interactions.

The utility of transcriptome analysis in the field was demonstrated in a whole blood RNA-Seq study of German cows aﬄicted with a novel disease termed bovine neonatal pancytopenia (Demasius et al., 2013). This fatal disease, for which no treatment is available, was observed in calves that ingested the colostrum of cows that were vaccinated against BVDV. The study revealed that the immune response occurring in the calves was due to the presence of a double stranded RNA that developed after vaccination with an inactivated single-stranded RNA. It also identified a novel previously uncharacterized gene that was upregulated during the immune response. In addition, 4,596 previously unknown transcribed loci were found. This helps illustrate the power and utility of gene sequencing which lies in its ability to provide novel information about genome annotation, especially for livestock species which trail behind human, mice or rat genomes in the quality of annotation (Andersson et al., 2015).

While we are still in the early stages of discovery of pathogen-directed host genome expression, it is not unreasonable to envision that different pathogens could elicit a host response profile that could be unique to a specific pathogen. This could be possible due to the fact that apart from a general immune response, a host could also detect a pathogen-specific molecular fingerprint, resulting in specific feedback interactions with the pathogen. Such information generated by the use of genomic tools could provide an opportunity to develop predictive and prognostic biomarkers for disease identification (especially in those lacking quality diagnostic measures) and treatment. Bhuju et al. (2012) used transcriptome sequencing to find IL-22 mRNA as a predictive biomarker of bovine tuberculosis vaccination efficacy in British calves, which was further confirmed by others (Aranday-Cortes et al., 2012). The authors commented that such predictive biomarker development in the rational design of vaccines would speed up the developmental procedure while reducing the cost of large animal experiments.

Similarly, next-generation sequencing can be used to gain insights into miRNA expression profiles as potential biomarkers. miRNAs have been shown to play important roles in regulating immunity and infection, a role that has been demonstrated in cattle (Glazov et al., 2009; Lawless et al., 2013). RNA-Seq was used to demonstrate that tuberculosis infection in cattle could be diagnosed using measured changes in the host transcriptome (Churbanov and Milligan, 2012; McLoughlin et al., 2014). Even more specifically, using a miRNA microarray, Golby et al. (2014) showed that expression of miR-155 could act as a biomarker of both tuberculosis disease development and the severity of the pathology.

The impact of the pathogen on the host microbiome and virome (and vice versa) provides another important avenue of investigation in cattle. Studies indicate that bovine gut microbiome composition can impact immune response. This can be achieved through bacterial secretion of anti-microbial compounds, or by influencing the expression of genes encoding host mucosal immune responses (Taschuk and Griebel, 2012). While commensal microbes have been documented to modulate host immune responses, studies investigating the impact of pathogen infection on the host health via perturbations of the microbiome are still sparse and warrant further investigation (Taschuk and Griebel, 2012; Ward and Guevremont, 2014). One such example involved a detailed analyses of lactic acid bacteria isolates from bovine mammary gland microbiota to select a candidate probiotic strain that could be used against infectious mastitis. The investigated properties included the ability to inhibit the growth of mastitis pathogens, the capacity to colonize host tissue and compete with mastitis pathogens, and the potential to stimulate the innate immune system. The genomes of five chosen lactic acid bacteria strains were fully sequenced to determine antibiotic resistance and inspect the genomic determinants of the characterized colonization and immunomodulation capacities. One strain, *Lactobacillus brevis* 1595, had no genes encoding for antibiotic resistance, and favorable genetic content coding for proteins involved in tissue colonization (Bouchard et al., 2015).

The use of emerging virome information to manipulate cattle microbial populations for positive economic impact was suggested (Ross et al., 2013b) and is supported by a study that showed that the phage community in an animal’s mucosa can provide a non-host-derived defense against invading bacteria (Barr et al., 2013). Viral metagenomic analysis indicated that the structural protein responsible for attaching phages to mucosa is enriched in viral communities associated with such an environment, and thus suggests a novel form of immunity that has not previously been recognized. Metagenomic analysis of microbiota has been used to predict complex phenotypes such as methane emission in cattle (Ross et al., 2013a); such an approach could also be used to predict disease phenotypes. Study of temporal variation of the host microbiome in relation to pathogen infection should also be considered. Analysis of bacterial and viral genomic diversity should aid in understanding how such variation can relate to animal robustness or susceptibility to infection and disease development.

## Treatment Development

Accumulated information on the etiology of diseases is necessary for the development of prophylactic and symptomatic treatments. An appropriate vaccination program has been shown to correlate with decreased morbidity and increased net value per animal due to enhanced product value and reduced treatment costs (Fulton et al., 2002). Although, development of more efficacious vaccines is believed to be an important contributing factor in decreased rates of BRD in the U.S., the ability of vaccines to adequately protect animals has been questioned, pointing to a need for novel strategies (Snowder et al., 2006; Theurer et al., 2015). In another case, sequencing of PI3V bovine isolates from U.S. identified strains that were previously thought to be foreign to this region. What was originally considered to be the only major strain turned out to compose only a minor fraction of the population, thus raising concerns about the efficacy of the currently marketed vaccines (Neill et al., 2015). Genomic study of the host interaction with the pathogen may provide further opportunities to design alternative strategies for protecting animals.

Knowledge of the pathogen genome allows for reverse genetic manipulation to discover functional roles of different genomic components and concomitant immunological host reactions. Such studies facilitate the discovery of important pathogen immunogenic characteristics. For example, a library of 115 synthetic peptides based on sequences of bovine leukemia virus genes was screened to discover novel epitopes able to activate cellular immunity in Japanese Black calves. Viral gp30 protein was identified as the best candidate for vaccine development (Bai et al., 2015). Armed with such information, genetic engineering of pathogen genomes can be undertaken to develop novel therapies, and sequencing can expedite the process of discovery. As the knowledge database expands, eventually a threshold of information will be obtained where modeling will substitute for experimental validation, allowing for faster development of therapeutic tools.

Such a systematic approach culminated in the development of a live virus BRSV vaccine that showed pronounced protection of calves from subsequent infection. Detailed analysis of immunogenic and functional characteristics of the virus was followed by genetic engineering to develop a recombinant virus with a deletion of one of its pathogenicity genes (Blodorn et al., 2014; Hagglund et al., 2014). An even more intricate approach was undertaken in the development of a novel BHV-1 vaccine where, based on past knowledge, the authors deleted three genes known to be responsible for anterograde neuronal transfer of the virus as well as avoidance of the host immune response. Compared to the vaccine currently mandated for use in the European Union, the new vaccine enhanced the protection of cattle from infection by significantly increasing the immune response of calves, while at the same time reducing the period and the amount of viral shedding after the challenge (Chowdhury et al., 2014). As additional information emerges about genes involved in the pathogenicity of different infectious agents, novel methodologies of vaccine development should continue to emerge. An example of progress in the field is the first *in silico* design of vaccines based on the sequencing of whole genomes of *H. somni* pathogens; the vaccines are currently being tested in animals (Madampage et al., 2015). Study of the variation in host vaccine response may also lead to new options for the selection of “vaccine-ready” animals.

In another example, BRSV infected Holstein calves treated with an experimental GS1 compound showed reduced viral load, disease symptoms, and lung pathology (Jordan et al., 2015). GS1 is a close structural analog of GS-5806, an inhibitor of the fusion between the viral envelope and the host cell membrane, and which recently was also shown as a potential successful treatment in humans (DeVincenzo et al., 2014; De Clercq, 2015). The antiviral properties of the compound were in part discovered by sequencing the envelope genes of virus isolates that exhibited reduced drug susceptibility to identify point mutations in the RSV *F* gene. Engineered virus recombinants with the same mutations acquired the resistance phenotype, pointing to the F fusion glycoprotein as the target of GS-5806 antiviral activity (DeVincenzo et al., 2014).

Another potential benefit of sequencing pathogens in the interest of treatment development is the possibility of identifying pathogens that could be utilized for whole organism vaccination. An example of such approach has already been mention with the use of co-infection with different strains of *T. parva* in sub-Saharan Africa (Woolhouse et al., 2015). It is conceivable that a proficiency of knowledge will be reached to allow genetic modification of pathogens of interest to create live whole organism vaccines that would not elicit severe disease symptoms but provide immunity against other more endangering pathogens.

Information about microbial genetics could also be an important driver of novel antibiotic production. The genetic background of antimicrobial resistance is poorly understood for many pathogens and the surveillance of antimicrobial drug resistance clearly indicates a loss of antibiotic susceptibility in common BRD pathogens (Portis et al., 2012; Rerat et al., 2012). Because microbial resistance can exhibit substantial in-herd variability, antibiotics are being administered to animals that cannot respond to treatment. An understanding of the genetic basis of microbial resistance would help curb unnecessary antibiotic use (Catry et al., 2005). This was demonstrated in Alberta feedlot calves for different BRD treatments: the value of metaphylactic antimicrobials varied in the net economic advantage by as much as $17.70 per treated animal depending on the drug choice (Van Donkersgoed et al., 2008a,b). Furthermore, a recent study of *M. haemolytica* isolates from Alberta cattle showed that BRD aﬄicted animals were more likely to harbor isolates with antibiotic resistance than healthy ones (37 vs. 2%, respectively; Klima et al., 2014a).

Whole genome sequencing of *P. multocida* (strain 36950) led to the finding of an ICE (a chromosome-derived mobile genetic element), the first of its kind identified in the species. It contained 88 genes including an astounding 12 different resistance genes which covered most of the antimicrobial agents approved for the control of BRD; three genes were identified that had never been observed previously in this bacteria (Michael et al., 2012a,b). Sequencing of the *M. haemolytica* (strain 42548) genome also led to the discovery of an ICE containing 107 genes, five of which conferred resistance to various antibiotics (Eidam et al., 2014). Significantly, these and other authors have demonstrated that ICE can be transferred from *M. haemolytica* and *H. somni* to *P. multocida* recipient cells, and vice versa, to confer the antimicrobial resistance phenotype (Michael et al., 2012b; Eidam et al., 2014; Klima et al., 2014b). A newly acquired sequence of *Fusobacterium necrophorum*, the causative agent of interdigital necrobacilosis (foot rot) in cattle, also contained a resistance-conferring ICE related to that of *Streptococcus pyogenes* (Calcutt et al., 2014). Such detailed knowledge can provide multiple advantages regarding animal treatment. It allows screening for pathogen multidrug resistance phenotypes and pointing to antibiotics that should not be provided to animals. It is also of vital importance to abate the dissemination of multiresistance into the broader environment; the spread of resistance reduces the effectiveness of prophylactic treatment, including zoonotic species with direct consequences to human health. Alternative approaches such as breeding for more robust animals to contribute to reduced untargeted or prophylactic antibiotic use are thus of key importance.

Gastro-intestinal nematodes are another example of an important group of pathogens that cause economic loss to the cattle industry (Stromberg and Gasbarre, 2006). Control by broad spectrum anthelmintic drugs has been very successful in the past, and meta-analysis of research trials identified parasite control as the most economically important pharmaceutical intervention used in U.S. beef production (Lawrence and Ibarburu, 2007). However, the production benefit is now beginning to erode due to the emergence of drug resistant parasites and new approaches to control are urgently needed (Stromberg and Gasbarre, 2006). Next-generation sequencing technologies offer new opportunities, from better identification of the types of pathogen present in order to tailor treatments, to understanding the genetic basis of parasite resistance and designing new drug approaches. This was well-demonstrated with recent publication of the *Haemonchus contortus* genome, the most widely studied nematode for anthelmintic resistance research and alternative treatment development. Analysis of the *H. contortus* gene expression network allowed the identification of five metabolic checkpoints as potential drug development targets. Two of these are currently under investigation in different species (Laing et al., 2013). Furthermore, the family of P-glycoprotein transporters, implicated in resistance of *H. contortus* to ivermectin and other anthelmintics, exhibit significant changes compared to other nematodes, information that could help elucidate the remarkable propensity to develop resistance by this parasite. Studies also show that the genetic differentiation between *H. contortus* and *H. placei* isolates (the more common parasite of cattle) demonstrates a high degree of genetic information exchange between the animal populations, including from different ruminant host species (Brasil et al., 2012).

## Breeding Programs

An important goal of the accumulated genomic information is undoubtedly the need to breed robust animals. Breeding programs based on genetic evaluations have been extremely successful, especially in the dairy sector where they have received the most attention, and their use has been adopted in a number of breeds around the world (Bouquet and Juga, 2013; Hutchison et al., 2014; Egger-Danner et al., 2015; Interbull, 2015). The rate of genetic progress measured in terms of a lifetime profitability of an animal is more rapid in the herds that use genomic testing than those that do not (Hutchison et al., 2014; Beavers and Van Doormaal, 2015). Recent data indicate that even in small cattle herds, limited by low reliability of breeding values, genomic breeding is still more economically and genetically superior to the conventional progeny-tested breeding schemes (Thomasen et al., 2014).

Past studies have shown that heritability estimates for disease incidence traits in cattle are usually low (Brotherstone et al., 2010; Schneider et al., 2010; Buttchereit et al., 2012; Parker Gaddis et al., 2014; Zare et al., 2014). However, moderate heritability was shown to be associated with general immune response to infectious diseases (Abdel-Azim et al., 2005; Thompson-Crispi et al., 2012b). It has also been pointed out that the genomic contribution to resistance has been found for many studied diseases, and could be even greater than the observed heritabilities due to factors such as incomplete exposure to infection or imperfect diagnosis (Bishop and Woolliams, 2014). Snowder et al. (2006) commented that years of greater BRD incidence allowed for more accurate heritability estimates because of an increased opportunity to observe the disease resistance phenotype. The authors proposed challenging cattle with specific pathogens so that the infection phenotype could be better understood from a molecular point of view. It has been also observed that pneumonic pasteurellosis in feedlot calves does not spread uniformly through feedlots, but is confined to specific pens. This suggests that mere exposure to a pathogen does not solely account for disease development; it is likely that calves’ phenotypic characteristics (including host-microbiome interplay) are important contributing factors (Rice et al., 2007). Together, these studies suggest that genetic variation could be exploited to breed livestock with more resistance to infectious diseases, or reduced infectivity. This is supported by models demonstrating that sire selection based on genetic merit can contribute to disease control (Van Hulzen et al., 2014). Evidence also suggests that breeding for disease resistance could be associated with improved reproductive performance and longevity in cows, further supporting this approach in the context of herd health management (Parker Gaddis et al., 2014).

These approaches are now being investigated in many studies and the first results are now available. The first GWAS of immune response traits in Canadian Holstein cattle demonstrated that significant SNP variation exists between high and low immune responders, and that the genetic profile could be used to breed animals of superior disease resistance and diminished infectivity (Thompson-Crispi et al., 2014b). Previous studies have shown that cattle classified as high immune responders have reduced incidences of diseases than low responder cows (Thompson-Crispi and Mallard, 2012; Thompson-Crispi et al., 2012a). Selection for animals with enhanced immunity has been shown to lead to advanced health of dairy cattle (reduced mastitis, improved response to vaccination and enriched colostrum quality) without compromising other important economic traits (Mallard et al., 2015). The fact that immune response is a measurable trait that can be used in breeding purposes has already been adopted by the Semex Alliance to identify superior dairy sires that are expected to be more profitable (Thompson-Crispi et al., 2014a; Mallard et al., 2015).

Investigations of specific disease resistance are also beginning to gain ground. GWAS, regional heritability and haplotype-sharing analyses identified two genes on chromosomes 2 (*MYO3B*) and 13 (*PTPRT*) associated with increased resistance to tuberculosis in Northern Ireland Holstein–Friesian dairy cattle (Bermingham et al., 2014). SNPs at these two loci accounted for 6.2 and 3.6% of variation, respectively, while SNPs in other genomic regions accounted for an additional 18.8%, pointing to the wide polygenic nature of resistance that could be used in breeding programs. Interestingly, a similar study in a different Irish population of same breed found that a different gene (*SLC6A6*) was associated with tuberculosis resistance (Finlay et al., 2012).

The Bovine Respiratory Disease Coordinated Agricultural Project aims to identify loci associated with BRD susceptibility and use this information to reduce the disease prevalence in beef and dairy cattle through selective breeding^1^. A GWAS identified SNPs that accounted for 20% of the variation in BRD incidence in dairy cattle, and 17–20% of the variation in clinical signs (Van Eenennaam et al., 2014). Moderate heritability estimates of 21% for either California or New Mexico populations strongly indicated that genomic selection could be used to reduce BRD prevalence in Holstein dairy cattle (Neibergs et al., 2014b). Further studies are being undertaken to uncover the causative SNPs, the role of CNV, the impact of pathogens on gene expression that contributes to disease development, as well as age influence on BRD susceptibility in different breeds (Neibergs et al., 2014b). The same group estimated that reducing incidence of BRD to 0.05% by breeding more robust dairy cattle could increase net margins by up to 16%, amounting to $1-2 million in annual savings in U.S. alone (Neibergs et al., 2014c; Van Eenennaam et al., 2014).

Another approach taken in analysis of heritability of pathogen-dependent BRD susceptibility was through a comparative analysis of bronchial lymph node transcriptomes of cattle challenged individually with different pathogens. In this way it was shown that the GWAS-identified QTL associated with a risk of BRD contained 142 genes differentially expressed between different pathogen insults. Furthermore, networks of gene expression allowed animals to be clustered together based on the pathogen involved (with exception of *M. bovis* and *P. multocida* which grouped together). This indicates that the genetic expression profile could serve as a potential predictive tool, which could be specific to a unique pathogen (Tizioto et al., 2015). To add to the emerging genomic research on BRD, GWAS studies on lung samples between case lesions and healthy control cattle, as well as incidence of persistent BVDV infection have also been presented (Casas et al., 2015; Keele et al., 2015). Both studies pointed to the complex genomic footprints behind these relevant phenotypes.

Perhaps the greatest progress has been achieved in studies toward mastitis resistance. Scandinavian countries have been using direct selection for mastitis resistance based on meticulous veterinary documentation of animal health status for many years (Sender et al., 2013). Genomic advancements have since led to identification of numerous SNPs in multiple candidate genes, in addition to detection of many QTL (Sender et al., 2013; Thompson-Crispi et al., 2014a). A sequence-based association study of clinical mastitis to refine previously detected QTL regions suggested *NPFFR2*, *SLC4A4*, *DCK*, *LIFR*, and *EDN3* as candidate genes for mastitis susceptibility (Wu et al., 2015b). Canadian Dairy Network now provides relative breeding values for the benefit of animal selection as of August 2014 (Jamrozik et al., 2013; Van Doormaal and Beavers, 2014). For example, sires chosen for best and worst values produced 3 vs. 21% of daughters with clinical mastitis in first lactation, and 9 vs. 28% in later lactations, respectively (Jamrozik et al., 2013). These are significant achievements which are expected to result in substantial financial savings. The latest analysis of mastitis costs in Ireland reveal that a 10% decrease in SCC (high values indicate that an animal is likely to be infected) equates to ∼$40 million in savings for both the dairy farm and the processing industry (Geary et al., 2013).

The relative ease with which whole genome sequence can be obtained also offers the opportunity to search for signatures of selection related to adaptations to different environments. This can include the development of resistance, decreased infectivity or tolerance to pathogens. For example, whole genome sequencing of only 14 Gir bulls (a *Bos indicus* breed from Brazil that is resistant to some tropical diseases of cattle) was able to identify loci under selection (Liao et al., 2013). One of the most striking selective sweeps identified several genes belonging to the cathelicidin gene family, such as *CAMP*, *CATHL1*, *CATHL2*, and *CATHL3*, which are potentially related to pathogen- and parasite-resistance.

While SNPs are the most frequent variants observed, genomic structural variants also contribute to phenotypic variation. Structural variants impact a higher percentage of sequence than SNPs, and can influence traits by altering gene expression dosage, or by exposing recessive alleles (Bickhart et al., 2012). For example, whole genome sequencing of 62 bulls from French Holstein, Montbéliarde and Normande breeds showed per animal an average of 199,200 small structural variations (< = 50 bp) and 305 large ones (>50 bp but frequently extending into 1000s of kb). Together, the deletions, duplications or inversions accounted for ∼15% of the genome with nearly 40% impacting gene-coding regions (Boussaha et al., 2015).

Importantly, studies demonstrate that these changes frequently affect genes related to immune response and host defense, and exhibit substantial intra-individual or between-breed variation (Liu et al., 2011; Stothard et al., 2011; Bickhart et al., 2012). In one such example, the *CIITA* gene, a trans-activator of the major histocompatibility complex class II receptor, was found to be duplicated in Angus cattle with nematode resistance (Liu et al., 2011). A deletion on chromosome 7 correlated with resistance in the same animal population was also described (Xu et al., 2014). Phenotypically, the key difference was that resistant cattle could better maintain inflammatory responses at the infection site than could parasite susceptible animals, and the authors hypothesized that the discovered CNVs could influence the expression of genes involved in such host resistance (Li et al., 2007). The shift in genomic focus marked by these studies will be instrumental in improving the robustness of animals to disease.

## Epigenetic Non-Mendelian Inheritance

Another area of research that is starting to gain more attention in cattle is imprinting, a form of epigenetic regulation that leads to preferential monoallelic expression of the genes of one parent over the genes of the other parent. It is not known how much phenotypic variation is due to epigenetic regulation. However, sequencing of epigenetic information of parental gametes, especially bull semen, can be expected to contribute important clues to an ideal breeding program related to offspring health.

One study uncovered a SNP in the maternally expressed imprinted bovine *NESP55* gene that was associated with SCC (Sikora et al., 2011). The authors suggested that the SNP is unlikely to have a causal effect (as it leads to the production of a different amino acid with similar biochemical properties), but it might be associated through linkage disequilibrium with other mutation(s). *RASGRF1* gene which has been shown to be imprinted in different species has also been associated with SCC in Irish Holstein–Friesian cattle (Magee et al., 2010). It has been shown that experimental application of pathogenic *Escherichia coli* (strain 1303) that caused acute mastitis in mid-lactating Holstein cows, coincided with the shutdown of αS1-casein expression due to increased promoter methylation, and a resulting chromatin condensation (Vanselow et al., 2006). These studies demonstrate that traits in which bacterial infection plays a part could also be under epigenetic regulation.

A recent study revealed that different cattle populations exhibit considerable SNP variability linked to epigenetic genes, further suggesting that DNA variants that modulate epigenetic processes is a potentially interesting area of research for livestock breeding purposes (Porto-Neto et al., 2014). Such knowledge has a potential to improve the accuracy of predicted breeding values, as current methods do not account for differences between the genetic and epigenetic merit of the resulting trait phenotype. While the probable impact of imprinting on phenotypic variance is speculative, the possibility of relatively large effects in cattle has been proposed (Meyer and Tier, 2012; Tier and Meyer, 2012).

Epigenetic studies might lead to an understanding of how environmental factors influence phenotypic traits, information that could then be incorporated into animal management. It is an exciting area of research awaiting full exploration, one that is likely to have a positive impact on predicting future animal health related phenotypes.

## Challenges

As can be expected from any novel disruptive technology, next-generation sequencing provides many challenges; the two most commonly cited are data complexity and utility cost. Continual investment in genomic studies will resolve these issues through further technological development, as evidenced by the steady rate of decreasing sequencing costs and emergence of available bioinformatics and computational methods. One approach to enhance the accuracy and power of the data analysis, is to use a combination of available software packages (Jin et al., 2014; Neibergs et al., 2014b). Another challenge facing the livestock industry will be the accuracy of the predicted breeding values, which will be dominated by two factors: reference population size and accuracy of measured phenotypes.

A large scale recording of established traits of economic importance needs to continue and must be extended to include a far broader assessment. It is particularly important to avoid compromising critical traits while pursuing improvement of others, as was the case with increasing milk production at the expense of retaining recessive defects. This was demonstrated in a particularly striking fashion in Nordic Red cattle with a 660 kb deletion on chromosome 12, and in Jersey cattle with a nonsense mutation in the *CWC15* gene on chromosome 15—both chromosome aberrations being associated with increased embryonic lethality—and maintained in a population at high frequency because they coincided with improved milk yield and composition (Sonstegard et al., 2013; Kadri et al., 2014). Remarkably, in the latter case, the deleterious mutation was propagated from a single ancestor and maintained in the population to a current carrier frequency of 23.4% (Sonstegard et al., 2013). Similarly, lack of complete phenotypic screening and a high level of inbreeding in the Holstein–Friesian breed led to outbreaks of bovine leukocyte adhesion deficiency and complex vertebral malformation, both of which could be traced to genetic defects in a single founder sire (Thomsen et al., 2006). In another example, cell-mediated immune response was shown to be positively associated with calving ease in heifers which was in turn negatively associated with antibody-mediated immune response. These two important types of immune response that target different types of pathogens, are often dichotomous in nature with antagonistic genetic correlation, and need to be mutually considered when selecting for phenotypes of interest (Thompson-Crispi et al., 2012b). The increased availability of whole genome sequencing information as illustrated by the 1000 Bull Genomes Project (Daetwyler et al., 2014) along with improved annotation (Andersson et al., 2015) presages a time when it will be possible to make predictions of such pleiotropic effects.

With regard to traits of robustness, apart from information delivered by sequencing, other phenotypic scores delivered by additional ‘omics’ (e.g., metabolomics), sensor-based or imaging technologies related to immune response or pathogen detection, will be of relevance in a bid to deliver more accurate diagnoses. One of the reasons for the low heritability of disease resistance is a difficulty in distinguishing healthy animals from sick animals. The accuracy of a heritability estimate is proportional to the accuracy to which a phenotype is measured (Van Eenennaam et al., 2014), including the microorganisms involved (Nash et al., 2000), thus the ability to correctly diagnose animal health status is of paramount importance to the progress of breeding healthier animals. Efforts of the Canadian Dairy Network to collect detailed health events by over 40% of national dairy producers allowed the development of genetic evaluations for mastitis resistance despite low heritability of this complex disease (Jamrozik et al., 2013; Van Doormaal and Beavers, 2014). A number of countries have adopted more detailed recording programs of health traits for genetic evaluations (Egger-Danner et al., 2015). Combining phenotype records from entities that process cattle information, from farms, veterinary labs, slaughter houses, dairy plants, and other relevant organizations, could enhance the development of more specific and precise breeding indexes (Egger-Danner et al., 2015). The International Committee for Animal Recording publishes a recording guidelines that include a comprehensive key of nearly 1000 diagnoses that can be used toward the genetic improvement of health traits in cattle (ICAR, 2014).

Genomic assisted breeding, also called genomic selection or genomic prediction, based on information from a large number of polymorphic markers distributed across the genome in individuals without phenotypes, was first suggested by Meuwissen et al. (2001). This has now been applied particularly successfully for dairy cattle (Bouquet and Juga, 2013; Hutchison et al., 2014). However, 1000s, if not 10s of 1000s of animals are required for accurate predictions, and this issue is even more daunting for beef cattle where many breeds of importance make it challenging to assemble large enough reference populations. For example, an increase in reference population from 2,253 to 11,756 Angus animal records augmented the accuracy of genomic prediction of EBVs (average correlation of 0.58 vs. 0.64), and explained an additional 18% of the genetic variance in the validation data (Boddhireddy et al., 2014). Potential solutions involve pooling animals from multiple regions, or even from different breeds, and using high density SNP panels to enable a wider probe for SNPs in linkage disequilibrium with causative mutations for genotyping (Lund et al., 2014; Wu et al., 2015b). Genotyping cows in addition to sires as another method of improving prediction accuracies of breeding estimates has been showing promising results (Hayes et al., 2014; Edel et al., 2015; Egger-Danner et al., 2015; Gao et al., 2015).

Sequencing animal genomes directly as opposed to genotyping would mean that genomic predictions would no longer be dependent on linkage disequilibrium between the panel SNPs and causative mutations, as these variants would automatically be present in the acquired data. Another added advantage of whole genome sequencing is that information is obtained on rare variants that contribute genetic variation but would typically be missed in a GWAS (Li et al., 2013). While this is not feasible for the entire reference population, cattle are typically relatively inbred. Sequencing of a few founder bulls that are responsible for siring 1000s of offspring would be sufficient for haplotype reconstruction and tracking of inheritance of traits of interest among the larger population. This was recently demonstrated with the sequencing of two such elite bulls (father and son) who sired more than 75,000 daughters between them, and account for ∼7% of all North American Holstein cow genomes, each (Larkin et al., 2012). The current 1000 Bull Genomes Project is spearheading this approach and is expected to improve the prediction accuracy of future genomic EBVs (Daetwyler et al., 2014; Hayes et al., 2014).

Another impact on genetic variance that might affect the accuracy of genomic predictions is the influence of epigenetic regulation and structural variation. However, studies of epigenetic regulation are difficult to execute with the current costs of whole genome methylation status sequencing, while what type of tissue is to be selected for such studies to be most informative is also an issue. Structural variations are also difficult to study as they are not amenable to easy identification with many next-generation sequencing platforms, or by genotyping.

Finally, the quality of cattle reference genomes requires further improvement to eliminate potential unassembled or misassembled regions with structural variations that can lead to loss of important functional understanding of the genome. Indeed, gaps in structural annotation of the reference genome was a reason provided by authors of the most recent sequenced genome of a Holstein cow to partially account for the large amount of novel variation described (Koks et al., 2014). Recent *de novo* reassembly of the unmapped resequenced DNA and RNA reads of the *Bos taurus* L1 Dominette 01449 reference genome identified additional bovine and other vertebrate species sequences, pointing to partially or completely missing or misassembled genes in the current reference assembly. A fraction of unmapped sequences also aligned with those of commensal or parasitic organisms, including as yet unsequenced or undiscovered nematode species (Whitacre et al., 2015).

One possible solution is to use sequencing platforms that generate very long sequence reads such as those of Pacific Biosciences Single-Molecule Real-Time sequencing technology which has been shown to rapidly and accurately resolve assembly of complex repetitive regions (Huddleston et al., 2014). A new bovine assembly Btau_5.0.1, based on the use of Pacific Biosciences sequencing, is now available in GenBank^2^. The challenge of the need of quality reference genomes can be extended toward additional species and specific breeds, however, international endeavors such as the 1000 Bull Genomes Project or the Canadian Cattle Genome Project are helping to alleviate these deficiencies (Daetwyler et al., 2014; Stothard et al., 2015).

Equally important is improving the annotation of gene function, if the utility of these new tools is to be fully exploited. Although, the bovine genome has been sequenced and published in Elsik et al. (2009), the accuracy of the genome annotation has shown modest progress with still >10% unannotated or improperly annotated sequences (Lewin et al., 2009; Zimin et al., 2009). An important step forward to bridge the genome-to-phenome gap is the creation of the FAANG (Andersson et al., 2015). The objective of FAANG is to integrate the emerging data for the purpose of annotating animal genomes, according to established protocol standards. Interested institutions that combine quality genome assemblies with phenotypic datasets can sign up at http://www.animalgenome.org/community/FAANG/index. Quality genomes with enhanced annotation of genetic function must be available for pathogens as well as the host, where the problems are not genome size but rather the huge number of genomes waiting to be analyzed.

Another option that may become available is the use of genetic modification and gene editing to create animals that are less susceptible to disease, technologies that allow improvements not possible through a traditional breeding approach. For example, transfer of bacterial genes into the cattle genome was done to produce resistance to certain strains of mastitis (Moore and Thatcher, 2006). Production of transgenic cows expressing human lysozyme in the milk was another recently reported method to achieve mastitis resistance (Liu et al., 2014). Homologous recombination was utilized to engineer tuberculosis-resistant cattle by addition of the *SP110* mouse gene previously implicated in inhibition of growth and multiplication of *M*. *bovis* (Wu et al., 2015a). A group led by Kemp at the International Livestock Research Institute plan to develop cattle that are resistant to trypanosomiasis (sleeping sickness in humans; Karaimu, 2014). The cattle will be modified with a gene from baboons that are naturally resistant to the disease (Thomson et al., 2009). Gene editing offers a new opportunity to utilize information on naturally occurring gene variants from next-generation sequencing projects (Proudfoot et al., 2015). One project seeks development of genetically dehorned cattle to overcome animal welfare issues associated with dehorning (Tan et al., 2013). It remains to be seen if this benefit will be sufficient to gain public support for the application of such technologies in animal production. Genetic manipulation of animals continues to be controversial in terms of food production, albeit this may change as the demand for animal protein increases or research succeeds in reducing incidence of zoonoses or positively impacting animal disease and health. Bruce et al. (2013) warn that the current system of regulation tends to close down such areas of application and innovation. They suggest that the focus should be on the product rather than the process used for its development, however, that argument has had little impact in terms of overcoming the opposition to genetic modification.

## Conclusion

Emerging scientific evidence demonstrates that next-generation sequencing fosters greater understanding of the genetic architecture behind complex phenotypes, including those of infectious disease. The disease phenotype can be further traced through accurate disease profiling, enhanced understanding of involved pathogens, and a molecular unraveling of the host–pathogen interaction—activities that collectively may lead to new ways of managing disease.

Already demonstrated applications are the diagnostic pathogen profiling, which can be used in herd management (such as reduced movement, isolation, choice of appropriate treatment), ability to develop diagnostic biomarkers to monitor disease progression, development of novel treatments based on an enhanced understanding of pathogen genomic architecture and its molecular impact on the host, as well as linking phenotypes of interest to specific loci for breeding purposes.

An example of adaptation of genome based information to future developments is the possibility of tissue specific pathogen based microarrays for genotyping on a routine scale, combined with the use of transcriptome biomarkers as predictors of disease phenotype. For farm use, nucleic acids based lab-on-a-chip biosensors for rapid pathogen identification can also be envisioned (Yoon and Kim, 2012). Genomic understanding of both commensals and pathogens could be used to manipulate host ecological and molecular environments to reduce the incidence of infection. This could even take the form of production of novel microorganisms in a combinatorial biosynthesis fashion. The emergence of genomics-based breeding programs targeting disease resistance and reduced infectivity will lead to higher quality animal produce and will have positive implications for human health in addition to the financial benefits for producers and consumers.

Genomic sciences can provide information about animals that was not available to previous generations of cattle producers. Sequencing tools will need to be implemented as part of multidisciplinary studies involving the producers who manage the livestock, veterinarians who collect appropriate samples, diagnostic laboratories that analyze the samples, research institutions that perform sequencing and interpret results, industrial stakeholders who disseminate the information, government participants who regulate the industry, as well as social scientists studying the barriers to the adoption of new technologies. Considering the potential of genomic sciences to improve herd well-being and alleviate the economic burden of cattle producers, we believe that next-generation sequencing has the potential to assist with the financial load of disease diagnosis and treatment to improve animal health and will have an important future in the cattle industry.

## Author Contributions

MR: substantial contributions to conception and design, data acquisition and analysis and writing of the manuscript; critical review for important intellectual content as a genetics and biochemistry expert; final approval of the version to be submitted; accountable for all aspects of the submitted work. LG: substantial contributions to analysis and interpretation of data as a bovine microbiome expert; minor contribution to writing of the manuscript; critical review for important intellectual content; final approval of the version to be submitted; accountable for all aspects of the submitted work. GP: substantial contributions to data acquisition and analysis and writing of the manuscript; critical review for important intellectual content as a livestock genomics expert; final approval of the version to be submitted; accountable for all aspects of the submitted work.

## Conflict of Interest Statement

The authors declare that the research was conducted in the absence of any commercial or financial relationships that could be construed as a potential conflict of interest.

## Footnotes

1 http://aux.ucdavis.edu/BRDcomplex/

2 http://www.ncbi.nlm.nih.gov/Traces/wgs/?val=AAFC05

## Abbreviations

BHV: bovine herpes virus
bp: base pair
BRD: bovine respiratory disease
BRSV: bovine respiratory syncytial virus
BVDV: bovine viral diarrhea virus
CNV: copy number variation
EBVs: estimated breeding values
FAANG: Functional Annotation of Animal Genomes Consortium
GWAS: genome-wide association studies
ICE: integrative conjugative element
kb: kilobases
MAP: *Mycobacterium avium* subsp. *Paratuberculosis*
miRNA: microRNA
PI3V: parainfluenza-3 virus
QTL: quantitative trait loci
RNA-Seq: RNA sequencing
SCC: somatic cell count
SNP: single nucleotide polymorphism
U.S.: United States
